# 丙烯海松酸键合硅胶固定相的制备及其在混合模式色谱分离中的应用

**DOI:** 10.3724/SP.J.1123.2024.10010

**Published:** 2025-07-08

**Authors:** Lei ZENG, Mengling WEI, Wei WEI, Hao LI, Bo’an SHI, Fuhou LEI

**Affiliations:** 1.广西民族大学化学化工学院，林产化学与工程国家民委重点实验室，广西林产化学与工程重点实验室，广西林产化学与工程协同创新中心，广西 南宁 530006; 1. Key Laboratory of Chemistry and Engineering of Forest Products of State Ethnic Affairs Commission，Guangxi Key Laboratory of Chemistry and Engineering of Forest Products，Guangxi Collaborative Innovation Center for Chemistry and Engineering of Forest Products，School of Chemistry and Chemical Engineering，Guangxi Minzu University，Nanning 530006，China; 2.湖北民族大学化学与环境工程学院，湖北 恩施 445000; 2. School of Chemical and Environmental Engineering，Hubei Minzu University，Enshi 445000，China

**Keywords:** 丙烯海松酸, 键合固定相, 混合模式色谱, acrylpimaric acid, bonded stationary phase, mixed-mode chromatography

## Abstract

本文以松香树脂酸衍生物丙烯海松酸为功能单体，通过开环反应将其键合到由*γ*-缩水甘油醚氧丙基三甲氧基硅烷修饰的烷基硅胶上，制备得到丙烯海松酸键合硅胶色谱固定相（Sil-APA）。傅里叶红外光谱、元素分析、Zeta电势分析和热失重分析等表征方法证明Sil-APA成功合成。由于Sil-APA固定相表面存在氢化菲环、羟基、羰基和羧基等功能基团，因此在分离过程中分析物与固定相之间会存在多种相互作用从而提升其分离性能。以疏水性、亲水性和有机碱性化合物为探针，通过研究流动相中有机相含量、pH和盐浓度对其保留性能的影响，证明Sil-APA固定相与分析物之间除疏水相互作用外，还具有亲水相互作用和弱阳离子交换作用，多重保留机制表明Sil-APA柱适用于混合模式液相色谱。Sil-APA柱对不同组分分析物具有良好的分离选择性进一步表明Sil-APA具有混合模式色谱性能，也证明其在复杂样品分析中具有良好的应用前景。此外，Sil-APA柱还具有良好的重复性（RSD为0.076%~0.356%，*n*=10）、稳定性（RSD为0.05%~0.193%，*n*=5）和重复制备性（RSD为0.498%~2.806%，*n*=4）。综上所述，本研究制备了一种基于丙烯海松酸改性硅胶色谱固定相，其在混合模式下展出的优异分离性能揭示了丙烯海松酸作为色谱固定相功能单体的可能性。该工作拓展了松香树脂酸在色谱分离领域的发展，也为在液相色谱领域开发基于天然产物改性硅胶的色谱固定相提供新的思路。

## Abstract (en)

The development of novel functional materials from renewable biomass resources has garnered widespread attention. This strategy not only effectively reduces the reliance on petrochemical raw materials in the preparation process， thereby reducing carbon emissions and mitigating environmental pollution， but also enhances their added value， promoting the development of related agriculture and forestry industries. In this study， we prepared acrylpimaric acid bonded on silica （Sil-APA） as a new stationary phase by linking the functional ligand to silica using *γ*-glycidoxypropyltrimethoxysilane as the silane coupling agent. The synthesized stationary phase was characterized by Fourier-transform infrared spectra （FT-IR）， elemental analysis （EA）， zeta potential analysis， and thermogravimetric analysis （TGA）. Acrylpimaric acid was successfully immobilized onto the surface of spherical silica via a ring-opening reaction involving the epoxy and carboxyl groups. Analytes and the stationary phase experience multiple interactions during the separation process owing to the coexistence of functional groups such as hydrogenated phenanthrene rings， as well as hydroxyl， carbonyl， and carboxyl groups on the surface of the Sil-APA stationary phase. Synergy involving multiple interaction mechanisms improves the separability and applicability of the Sil-APA stationary phase. We used hydrophobic， hydrophilic， and ionic compounds to probe the effects of organic-phase content， pH， and buffer-salt concentration in the mobile phase on the chromatographic performance of the Sil-APA column. The logarithm of the retention factor （log *k*） for alkylbenzenes in the stationary phase was found to decrease with increasing methanol content in the mobile phase during reverse-phase liquid chromatography （RPLC）； hence， the Sil-APA stationary phase exhibited typical reverse-phase retention behavior. The Sil-APA column exhibited stronger aromatic selectivity and weaker hydrophobic selectivity compared to a C18 column， which enables polycyclic aromatic hydrocarbons （PAHs） to be more-strongly retained than alkylbenzenes while also separating these hydrophobic compounds in less time. The ln *k* for nucleosides/bases on the stationary phase decreased with increasing water content in the mobile phase in hydrophilic interaction liquid chromatography （HILIC） mode， confirming that the Sil-APA column has typical hydrophilic retention characteristics for the separation of nucleosides/bases. Furthermore， the relationship between the pH and buffer-salt concentration of the mobile phase and ln *k* for nucleosides/bases reveals that hydrogen bonding and electrostatic interactions between the stationary phase and the analytes play important roles in addition to hydrophilic interactions. The ln *k* for ionic compounds was observed to decrease with increasing buffer-salt concentration in the mobile phase in ion-exchange chromatography （IEC） mode， while concurrently increasing with the pH of the mobile phase. These results demonstrate that the Sil-APA column exhibits classical cation-exchange behavior for the separation of cationic compounds. These multiple retention mechanisms render the Sil-APA column suitable for mixed-mode liquid chromatography （MMC）. The Sil-APA column exhibits good separation selectivity for different analyte components， further demonstrating its mixed-mode chromatographic performance and potential for complex sample analysis. Furthermore， Sil-APA demonstrated excellent chromatographic repeatability， stability， and reproducibility， as evidenced by low retention-time relative standard deviations （RSDs） of 0.076%–0.356% （*n*=10）， 0.05%–0.193% （*n*=5）， and 0.498%–2.806% （*n*=4） for inter-day， inter-day， and batch-to-batch precisions， respectively. In summary， we designed and prepared an acrylic-acid-modified silica stationary phase material for use in liquid-chromatography applications. The excellent separation performance observed under mixed-mode conditions demonstrates that acrylpimaric acid can possibly be used a functional stationary-phase monomer， thereby potentially broadening the applications scope of resin acids in separation science. The facile preparation of Sil-APA and its promising applications scope may also provide new concepts for the development of stationary-phase materials based on natural-product-modified silica in the liquid-chromatography field.

高效液相色谱（high performance liquid chromatography，HPLC）作为一种高效快速的分析分离技术，已被广泛应用于生命科学、化学化工、食品安全和环境监测等领域^［1-4］^。固定相作为HPLC的关键组成部分，对HPLC的分离模式以及分析物的洗脱行为具有决定性影响^［5］^。因此，针对不同的分离需求可自行选择相对应的分离模式，例如适用于疏水化合物分离的反相液相色谱（reversed-phase liquid chromatography，RPLC）、适用于亲水化合物分离的亲水相互作用色谱（hydrophilic interaction liquid chromatography，HILIC），以及适用于离子型化合物分离的离子交换色谱（ion-exchange chromatography，IEC）。由于单一分离模式的HPLC具有较强的适用性，因此其依旧占据着色谱分析领域的主导地位。然而，在面对复杂样品的分析分离时，单一分离模式的HPLC则存在一定的局限性，RPLC不适用于分离极性化合物和亲水性化合物；HILIC则仅适用于分离极性化合物和亲水性化合物；IEC仅对离子化合物具有较好的分离性能；而混合模式色谱（mixed-mode liquid chromatography，MMC）可以有效地解决单一分离模式色谱应用受限的问题，其通过固定相表面不同化学性质的功能基团与分析物的多种相互作用，实现多种保留机制，从而应对复杂样品的分离^［6-8］^。目前，多种保留机制相结合的混合模式色谱固定相已经被开发报道。Wang等^［9］^通过“巯-烯”点击反应将半胱氨酸键合到乙烯基功能化硅胶表面，再使用溴代烷烃对半胱氨酸的氨基进行季铵化修饰，制备了4种RPLC/IEC混合模式半胱氨酸功能化的两性离子色谱固定相，并将其应用于蛋白质的分离。Fan 等^［10］^以4-乙烯基吡啶和*N，N*-二甲基丙烯酰胺为功能单体，氰酸三烯丙酯作为交联剂，通过“巯-烯”点击反应构建一种含氮的非共轭柔性网络改性硅胶混合模式色谱固定相Py-TAC-DMA@SiO_2_，该固定相具有良好的RPLC/HILIC混合保留机制。王晓庆等^［11］^通过自由基聚合将聚（苯乙烯-丙烯酸）共聚物键合到硅胶上，制备得到一种反相/亲水/阳离子交换（RPLC/HILIC/ICX）混合模式色谱固定相SiO_2_@P（St-b-AA），该固定相既可在RPLC模式下分离烷基苯、多环芳烃等疏水性化合物，又可在HILIC/ICX混合模式下同时实现极性小分子和阳离子型化合物的分离。Liu等^［12］^通过在ZIF-67@SiO_2_表面修饰一层由丙烯酸和*N，N*-二甲基丙烯酰胺组成的两亲性聚合物水凝胶构建了一种ZIF-67/水凝胶混合模式的色谱固定相，并将其应用于化妆品中烟酰胺和环境内分泌干扰物邻苯二甲酸酯的分离检测。虽然许多研究工作已经建立起多种构建混合模式色谱固定相的方法，但开发具有更高分离效率、更广泛适用性和更低成本的混合模式固定相对满足复杂样品分离分析需求具有重要意义。

随着全球石化资源日益匮乏、气候变暖等环境问题不断加剧，环境保护与能源需求的平衡已成为全球性的挑战。为实现可持续发展的美好愿景，生物质作为一种来源广泛的可再生资源，其应用前景受到广泛关注。利用生物质资源为原料合成各种精细化学品和生物基功能材料成为目前研究和发展的热点^［13，14］^，部分生物质材料因其独特化学结构与性能在新型色谱固定相的设计与制备展现出良好的应用潜能^［15-17］^。松香作为一类由松树分泌的天然树脂，其主要成分为松香树脂酸，是我国的特色林产资源之一^［18］^。因其绿色可再生，且具有良好的疏水性、刚性和生物相容性，已被广泛应用于构建新型疏水材料^［19-21］^和反相液相色谱固定相^［22-26］^。丙烯海松酸（acrylpimaric acid，APA）作为一种重要的松香衍生物，是由丙烯酸和松香通过 Diels-Alder 反应制备，其结构除氢化菲环骨架外还含有两个羧酸基团^［27］^。氢化菲环具有良好的疏水性，而羧酸基团除了提供亲水作用外，其解离后还可以提供阳离子交换作用。因此，将丙烯海松酸作为功能单体接枝到硅胶上可能是一种制备混合模式液相色谱固定相的有效方法。此外，文献调研发现目前还没有关于将松香树脂酸及其衍生物直接作为功能单体用于构建混合模式色谱固定相材料的报道。

因此，本文提出了一种以丙烯海松酸构建混合模式色谱固定相的策略。以丙烯海松酸为功能单体，*γ*-缩水甘油醚氧丙基三甲氧基硅烷（GPS）为硅烷偶联剂，通过开环反应将其键合到硅胶上制备得到丙烯海松酸键合硅胶色谱固定相（Sil-APA），并利用一系列分析测试方法对其进行表征。分别以疏水性化合物、亲水性化合物和阳离子化合物为分析物测试其在反相、亲水和阳离子交换模式下的分离性能，并对其保留机制进行研究。通过分离不同极性组分的混合物进一步考察Sil-APA的混合模式分离性能，并与C18和Amide柱进行对比。此外，还对Sil-APA柱的分离重复性、稳定性和可重复制备性进行考察了。

## 1 实验部分

### 1.1 仪器与试剂

NicoletiS10傅里叶变换红外光谱仪（美国Nicolet公司）；TGA/DSC3+同步热分析仪（瑞士Mettler-Toledo公司）；Vario EL cube 元素分析仪（德国Elementar公司）；Zetasizer Nano ZS纳米粒度电位仪（英国Malvern公司）；全自动比表面及微孔分析仪（美国Micromeritics公司）；LC-15C型高效液相色谱仪，SPD-15紫外检测器和SIL-16自动进样器（日本Shimadzu公司）； Sartorius arium pro超纯水机（德国Sartorius公司）；Z0050716装柱机（美国Scientific Systems公司）。

APA（95%，实验室自制^［28］^）；硅胶（粒径：5 μm， 孔径：10 nm，比表面：360 m^2^/g，苏州纳微科技公司）；不锈钢色谱柱管（250 mm×4.6 mm，大连依利特分析仪器有限公司）；Eclipse Plus C18色谱柱（250 mm×4.6 mm，5 μm，美国安捷伦公司）；XAmide 色谱柱（250 mm×4.6 mm，5 μm，浙江华谱新创科技有限公司）；GPS（97%）、苄基三乙基氯化铵（TEBAC，98%）、乙酸铵、乙酸、氨水、烷基苯类化合物、多环芳烃类化合物、苯甲酸类化合物和黄酮类化合物（分析纯，上海阿拉丁生化科技股份有限公司）；碱基核苷酸类化合物、咖啡因、可可碱、阿米替林、去甲替林和普萘洛尔（分析纯，上海麦克林生化科技股份有限公司）；甲醇和无水乙醇（分析纯，成都西陇化工有限公司）；甲醇和乙腈（色谱纯，美国赛默飞世尔科技公司）。

### 1.2 丙烯海松酸键合硅胶色谱柱的制备

#### 1.2.1 固定相的制备

丙烯海松酸键合硅胶固定相的制备过程如图1所示。首先将硅胶按照先前工作中的实验步骤进行活化处理^［17］^。称取6.00 g活化硅胶置于圆底烧瓶中，依次加入6 mL GPS和80 mL无水甲苯，在N_2_保护下搅拌使其完全分散，再升温至110 ℃反应24 h。待反应完毕冷却至室温后依次用丙酮、甲醇和无水乙醇对产物进行洗涤，放入真空干燥箱80 ℃干燥12 h，制备得到环氧硅胶（Sil-GPS）。称取3.50 g丙烯海松酸置于50 mL无水甲苯中，升温至80 ℃搅拌0.5 h使其完全溶解。然后依次向反应体中加入5.00 g Sil-GPS和0.05 g TEBAC（催化剂），搅拌使其完全分散，在N_2_保护下升温至110 ℃反应24 h。待反应完毕趁热收集产物并依次用乙酸乙酯和无水乙醇洗涤，在60 ℃下真空干燥12 h，得到Sil-APA。

**图1:**
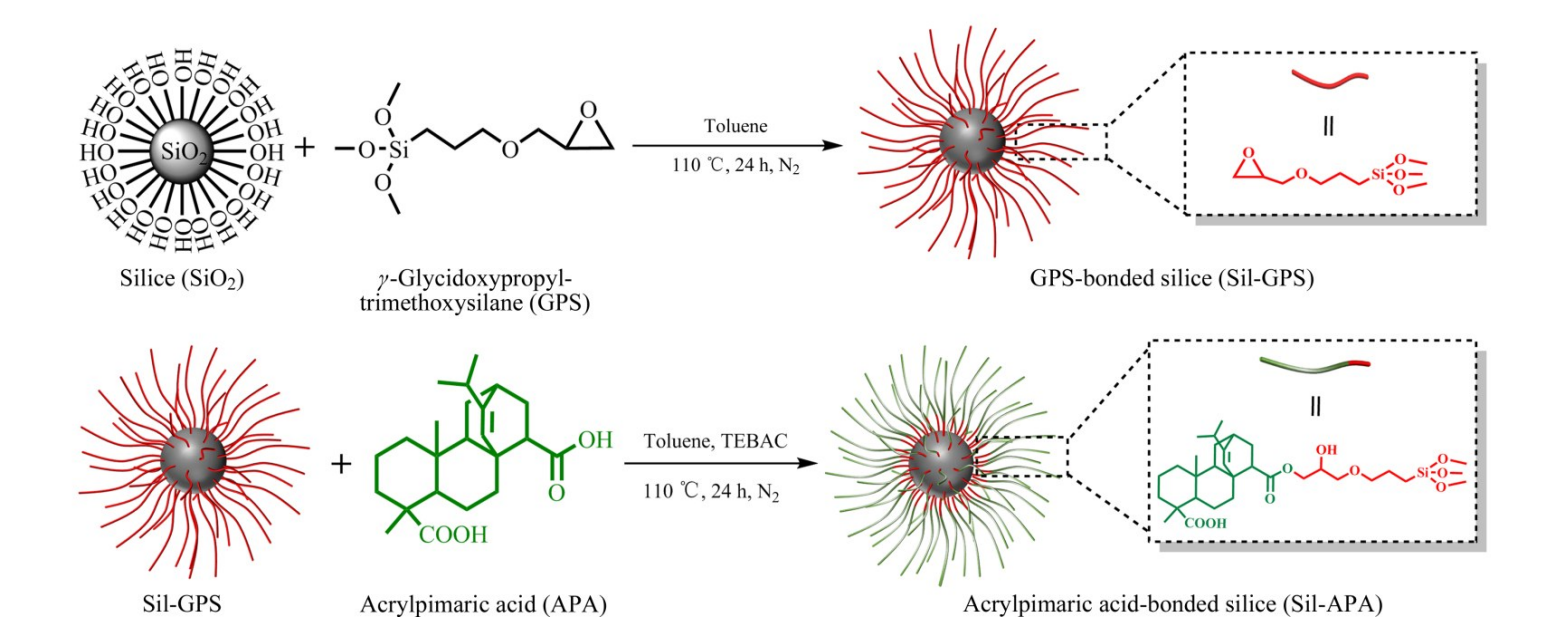
丙烯海松酸键合硅胶色谱固定相的制备

#### 1.2.2 装柱

采用高压匀浆填充法将Sil-APA固定相装填到不锈钢柱管中（250 mm×4.6 mm）。称取4.00 g固定相置于烧杯中并加入60 mL异丙醇-三氯甲烷（1∶1，v/v），超声使其分散均匀。在超声5 min后，将制备的匀浆液装入匀浆罐中，以甲醇为顶替液，在45 MPa的压力下装填30 min，装填完毕后以甲醇为流动相冲洗装，备用。实验中所使用的活化硅胶柱采用相同的方法装填，装填完毕后以乙腈为流动相冲洗，备用。

### 1.3 色谱条件

分离烷基苯和多环芳烃采用甲醇-水（70∶30，v/v）为流动相；分离黄酮类化合物采用甲醇-0.05%磷酸水溶液（60∶40，v/v）为流动相；分离碱基/核苷采用乙腈-50 mmol/L乙酸铵水溶液（91∶9，v/v）为流动相；分离碱性化合物采用乙腈-50 mmol/L乙酸铵水溶液（pH=5.0）（40∶60，v/v）为流动相；分离苯胺类化合物采用乙腈-50 mmol/L乙酸铵水溶液（pH=4.5）（40∶60，v/v）为流动相；分离混合化合物采用乙腈-50 mmol/L乙酸铵水溶液（pH=5.0）（50∶50，v/v）为流动相。流速均为1.0 mL/min，采用等度洗脱；柱温均为30 ℃；检测波长均为254 nm；进样量均为5 μL。

## 2 结果与讨论

### 2.1 丙烯海松酸键合硅胶固定相的表征

采用元素分析、傅里叶红外光谱、热失重分析和Zeta电势分析对制备的固定相材料进行表征。采用Vario EL cube元素分析仪对SiO_2_、Sil-GPS和Sil-APA中的C和H元素的含量进行测定，结果如表1所示。相较于SiO_2_，Sil-GPS中的C和H元素的含量分别增加到4.365%和1.042%，表明*γ*-缩水甘油醚氧丙基三甲氧基硅烷成功键合到SiO_2_上，其键合密度为1.642 μmol/m^2^。Sil-APA中的C和H元素的含量分别增加到8.114%和1.317%，表明丙烯海松酸通过环氧开环反应接枝到Sil-GPS上，成功制备得到Sil-APA固定相材料，其键合密度为0.521 μmol/m^2^。相较于Sil-GPS，Sil-APA的键合密度较小，这可能是由于丙烯海松酸具有较大的空间构型，已键合到硅胶上的丙烯海松酸会对未反应的环氧基形成了屏蔽效应，从而影响其键合密度。

**表1:** 硅胶、环氧硅胶和Sil-APA固定相的元素分析结果

Analyte	Elemental contents/%		Surface coverage/（μmol/m^2^ ）
	C	H	
SiO_2_	0.723	0.343	-
Sil-GPS	4.365	1.042	1.642
Sil-APA	8.414	1.317	0.521

SiO_2_、Sil-GPS和Sil-APA的傅里叶红外光谱如图2所示，相较于SiO_2_，Sil-GPS在3 467 cm^-1^处的Si-OH伸缩振动峰明显减弱，976 cm^-1^处的Si-OH弯曲振动峰明显消失；并且在908、2 956和2 880 cm^-1^处分别出现环氧基的特征峰和-CH_2_-伸缩振动峰，表明 GPS成功修饰到SiO_2_上。Sil-APA在908 cm^-1^处的环氧基特征峰消失，2 956 cm^-1^和2 880 cm^-1^处的吸收峰明显增强，并且在1 719 cm^-1^处出现C=O的伸缩振动峰，表明成功将丙烯海松酸接枝到Sil-GPS上，制备得到Sil-APA固定相材料。

**图2:**
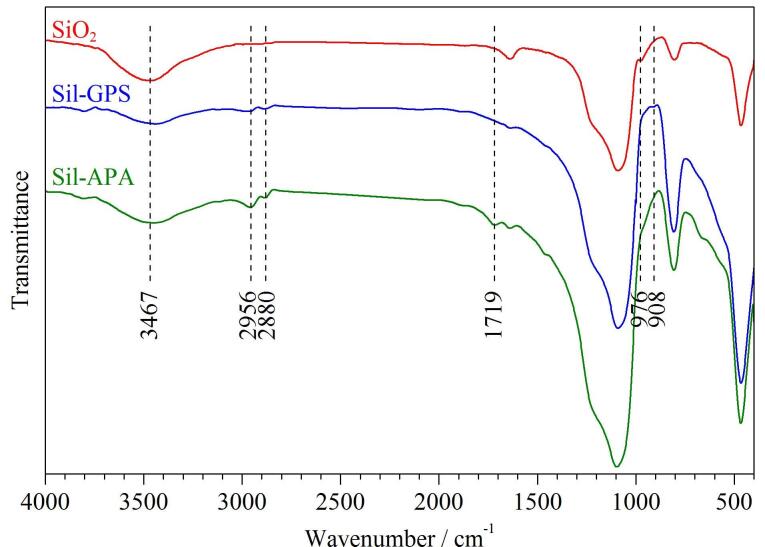
硅胶、环氧硅胶和Sil-APA的红外光谱图

通过热失重分析对固定相Sil-APA的热稳定性进行评价，如图3所示。3种测试材料在25~120 ℃处出现约1.2%的失重现象，这是测试材料脱水造成的。Sil-GPS在300 ℃处开始出现明显的热失重现象；而Sil-APA分别在200 ℃处和300 ℃处出现较明显的热失重现象，该结果表明丙烯海松酸的热分解温度约在200 ℃，而偶联剂的热分解温度约在300 ℃。在800 ℃时，SiO_2_、Sil-GPS和Sil-APA的质量损失分别为3.28%、7.52%和11.41%。上述实验结果不仅表明成功制备得到Sil-APA固定相，还证明该固定相具有良好的热稳定性。

**图3:**
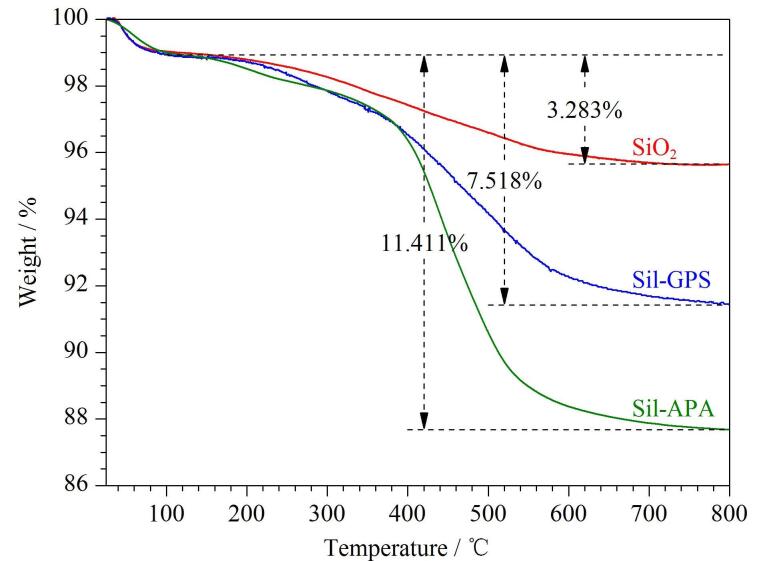
硅胶、环氧硅胶和Sil-APA的热失重曲线图

由于Sil-APA固定相上存在羧基，在pH>4.5 时，羧基会解离成羧酸根，使固定相带负电荷。为了考察固定相所带电荷情况，对固定相Sil-APA进行Zeta电势测定。称取Sil-APA固定相10 mg，超声分散于10 mL去离子水中制备得到Sil-APA固定相悬浮液。取3份1 mL的固定相悬浮液置于10 mL离心管中，分别加入7 mL pH为3.0、5.5和8.0的20 mmol/L的乙酸铵水溶液，超声分散均匀后将固定相悬浮液注射到样品池中在25 ℃进行Zeta电位测试。在3个测试条件下，Sil-APA的Zeta电势别为-0.404、-9.614和-22.725 mV。随着测试溶液pH的增加，Sil-APA的Zeta 电势逐渐降低，并呈现负值，表明此时Sil-APA固定相带负电荷。Zeta电势分析结果表明Sil-APA固定相不仅被成功合成，并且有望用于阳离子型化合物的分离，其分离性能可以通过调节流动相的pH进行调控。

### 2.2 Sil-APA色谱柱分离性能评价

#### 2.2.1 反相分离模式

Sil-APA固定相上既有疏水氢化菲环，还有能形成 *π-π* 相互作用的功能基团羰基^［29］^。因此，研究Sil-APA柱在反相模式下的分离性能十分必要。首先，我们选用6种烷基苯来考察Sil-APA柱的分离性能和保留行为，并与C18柱进行对比。烷基苯的保留因子对数（log *k*）与其油水分配系数（log *P*_o/w_）的关系如图4a所示。随着流动相中甲醇体积分数的增加，各烷基苯的log *k* 值均逐渐减小，符合反相模式分离特征；并且不同色谱条件下，各烷基苯的log *k*与其log *P*_o/w_之间均呈现出良好的线性关系，表明烷基苯在Sil-APA色谱柱上的分离机制是疏水相互作用。在相同的色谱条件下，Sil-APA和C18柱对6种烷基苯均实现基线分离（图4b和附表1，https://www.chrom-China.com），且洗脱顺序完全一致。但相较于C18柱，Sil-APA柱在10 min内就完成了烷基苯的洗脱分离过程，这是由于Sil-APA固定相上存在极性官能团，削弱了分析物与固定相之间的疏水相互作用，从而实现非极性化合物更快的洗脱分离。

**图4:**
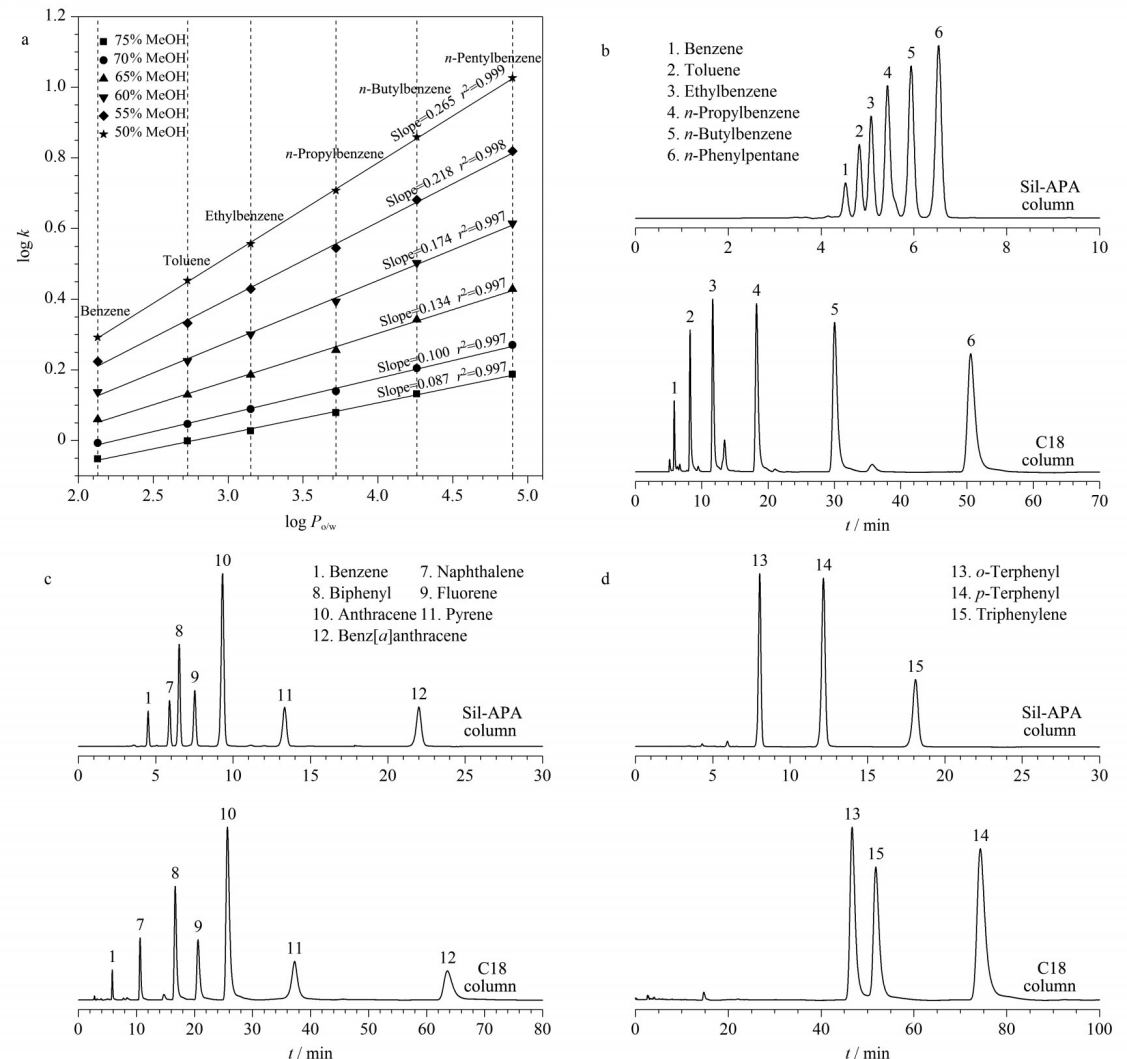
疏水性分析物在反相模式下的分离性能及保留机制评价

多环芳烃（PAHs）作为一类广泛存在于环境中的有机污染物，不仅会对生态系统造成危害，还会通过食物链进入人体，对人体健康造成威胁，因此对其进行准确的分析检测十分必要。为了考察Sil-APA对PAHs的分离性能，以苯和6种PAHs为分析物测试其分离性能并与C18柱进行对比。两根色谱柱对这7种物质均表现出良好的分离性能且洗脱顺序一致（图4c和附表2），表明疏水相互作用在分离过程中起主导作用。由于Sil-APA柱的疏水性弱于C18柱，因此在相同的色谱条件下Sil-APA柱可以在更短的时间内实现苯和PAHs化合物的洗脱分离，且色谱峰峰形更加对称。然而值得注意的是，相较于C18柱对分析物的保留性能与其疏水性呈正相关而言，Sil-APA柱对PAHs的保留性能强于烷基苯。这是由于PAHs中的苯环结构能与Sil-APA固定相表面的羰基形成 *π-π* 相互作用，从而增强Sil-APA柱对PAHs的保留性能，这也表明PAHs在Sil-APA柱上的成功分离是疏水相互作用和 *π-π* 相互作用协同作用的结果。此外，通过分离邻三联苯、对三联苯和三亚苯（图4d），进一步研究 *π-π* 相互作用对Sil-APA色谱柱分离性能的影响。相较于C18柱上的洗脱顺序按其疏水性排列，Sil-APA对疏水性较弱但具有平面构型的三亚苯具有更强的保留性能。同非平面构型的邻三联苯和对三联苯相比，平面构型的三亚苯具有更强的共轭体系能够与Sil-APA固定相形成更强的 *π-π* 相互作用，从而增强其在色谱柱上的保留性能^［30］^。根据Tanaka 测试^［31，32］^，利用三亚苯与邻三联苯的分离因子*α*_T/O_（*α*_T/O_ = *k*_triphenylene _/*k_o_*_-terphenyl_）评价色谱柱的平面选择性；利用邻三联苯与正戊基苯的分离因子*α*_O/PB _（*α*_O/PB_ = *k_o_*_-terphenyl_/*k_n_*_-pentylbenzene_）评价色谱柱的芳香选择性。Sil-APA柱的 *α*_T/O _（2.75）和 *α*_O/PB _（1.36）值均大于C18柱（*α*_T/O _=1.12，*α*_O/PB _=0.92），该结果表明相较于C18柱，Sil-APA柱具有更强的形状选择性和芳香选择性。

将烷基苯与PAHs的log *k*值分别进行线性拟合，进一步比较Sil-APA与C18对这两类化合物的保留性能（图5）。相较于两根色谱柱上烷基苯的log *k*值之间呈现良好的线性关系（*r* ^2^=0.997），PAHs的log *k*值之间的线性关系相对较弱（*r* ^2^=0.984），尤其是非平面构型的PAHs（*r* ^2^=0.841），表明烷基苯在Sil-APA柱上的保留机制与C18柱类似，以疏水作用为主；而PAHs在两根色谱柱上的保留机制则存在较大差异，这是因为Sil-APA柱对PAHs的保留机制除疏水作用外，还具有*π-π*相互作用。平面构型PAHs线性方程的斜率（0.840）大于非平面构型PAHs（0.483），并且平面构型与非平面构型PAHs线性方程的斜率均大于烷基苯（0.238），该结果进一步验证*π-π*相互作用增强了Sil-APA柱的芳香选择性和立体选择性，使其对PAHs具有更强的保留性能，尤其是对具有更大共轭体系的平面构型PAHs^［30］^。

**图5:**
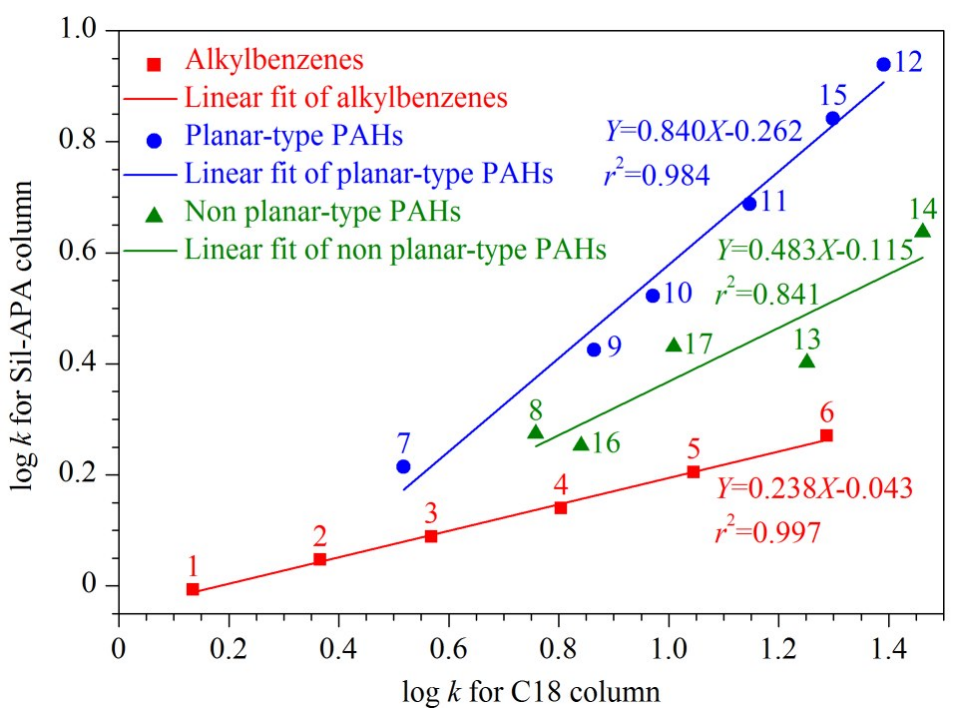
C18与Sil-APA柱上疏水性分析物log *k*值的关系图

Sil-APA固定相表面存在大量的羧基和羰基基团，这两种基团均是良好的氢键作用位点。为了研究氢键相互作用对Sil-APA柱分离性能的影响，以8种黄酮类化合物（图6a）作为分析物测试Sil-APA柱的分离性能，并与C18柱进行对比。由图6b可以观察到，Sil-APA柱和C18柱对这8种黄酮类化合物均表现出良好的分离性能，但这8种黄酮类化合物在两根色谱柱上保留性能存在着明显差异。在Sil-APA柱上除槲皮素（峰5）、桔皮素（峰7）和山柰素（峰8）外，其余黄酮类化合物的洗脱顺序与C18柱一致，按其疏水性排列。相较于金雀异黄酮（3个-OH），Sil-APA柱对疏水性相对较弱但结构中具有更多羟基基团的槲皮素（5个-OH）表现出更强的保留性能，一方面是由于槲皮素具有交叉共轭体系会与固定相形成更强的*π-π*相互作用，另一方面更多的羟基基团也会与固定相形成更强的氢键相互作用。相较于桔皮素（0个-OH），Sil-APA 柱对山柰素（3个-OH）具有更强的保留性能也验证了氢键相互作用对其分离性能的影响。尽管黄酮类化合物与Sil-APA固定相之间存在 *π-π* 相互作用和氢键相互作用，但槲皮素（5个-OH）、山柰酚（4个-OH）和桔皮素（0个-OH）在Sil-APA柱上的洗脱顺序依旧按其疏水性排列，该结果表明在分离过程中 *π-π* 相互作用和氢键相互作用有一定影响但疏水相互作用仍占据主导地位，另一方面也表明Sil-APA柱对黄酮类化合物的成功分离是疏水、*π-π* 和氢键作用协同的结果。

**图6:**
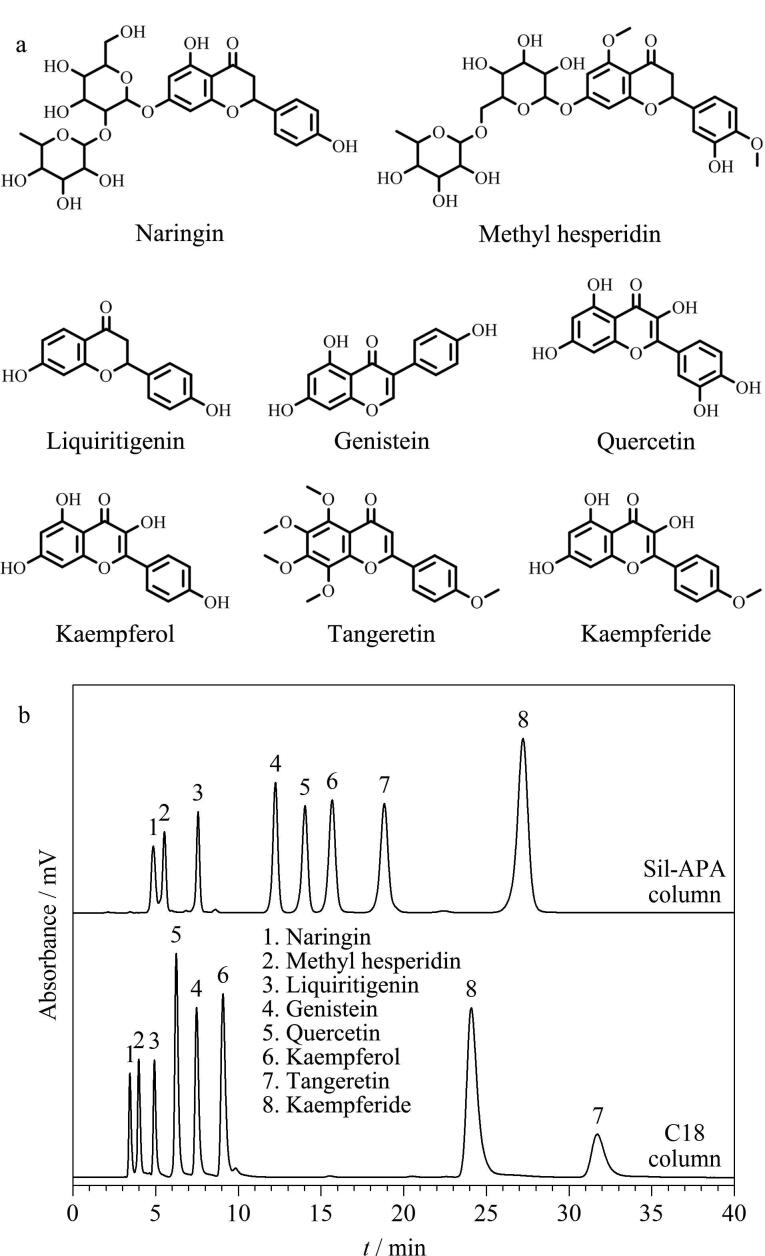
黄酮类化合物的（a）结构式及其（b）分离色谱图

#### 2.2.2 亲水分离模式

Sil-APA固定相上大量的亲水基团（-COOH和-OH）可以为极性溶质提供亲水相互作用位点。本节选择用8种碱基/核苷来考察Sil-APA柱在亲水模式下的分离性能及其保留机制。为测试Sil-APA柱在HILIC模式下的分离性能，以8种碱基/核苷为分析物测试其分离性能，并与活化硅胶柱和XAmide柱进行对比。在高有机相比例的洗脱液中，8种碱基/核苷在Sil-APA柱上成功实现基线分离，表明Sil-APA柱具有良好的亲水色谱性能（图7a和附表3）。而在相同的色谱条件下活化硅胶柱和XAmide柱无法实现碱基/核苷的基线分离，并且XAmide柱需要更长的时间才能将碱基/核苷完全洗脱（附图2）。值得注意的是碱基/核苷在Sil-APA柱上的洗脱顺序与活化硅胶柱和XAmide柱存在明显的差异，表明8种碱基/核苷在3根色谱柱上的分离机制有所不同。在HILIC模式下，硅胶柱的保留性能主要受Si-OH影响，氢键作用的强度决定了碱基/核苷的洗脱顺序^［33］^。然而，APA功能单体引入可以在一定程度上屏蔽残留的Si-OH基团提供的氢键作用，导致键合单体所提供的作用力成为主导，从而造成不同的洗脱顺序。这也从化学表征的角度证明Sil-APA的制备是成功的。此外，8种碱基/核苷的ln *k*值随流动相中水体积分数的增加而减少（图7b），符合亲水模式分离特征，但8种碱基/核苷在Sil-APA柱上的洗脱顺序与其极性强度存在差异，这也进一步表明8种碱基/核苷在Sil-APA柱上的保留机制除亲水相互作用外还存在其他作用。通过使用多模式保留模型（公式1）进一步研究碱基/核苷在Sil-APA柱上的保留机制^［34］^：


$$lnk=a+blnC_{B}+cC_{B}$$ (1)

其中*a*为常数，*b、c*分别为ln *C*_B _和*C*_B_的系数，*C*_B_为流动相中水的体积分数。公式（1）对8种碱基/核苷的ln *k*随流动相中水含量的变化关系具有良好的拟合效果（*r* ^2^=0.997 8~0.999 8，见附表4），这也表明Sil-APA在亲水模式下的保留机制不仅受固定相表面富水层与流动相之间的亲水分配作用的控制，还可能涉及表面吸附的相互作用，如 *π-π*、氢键和静电引力等相互作用。

**图7:**
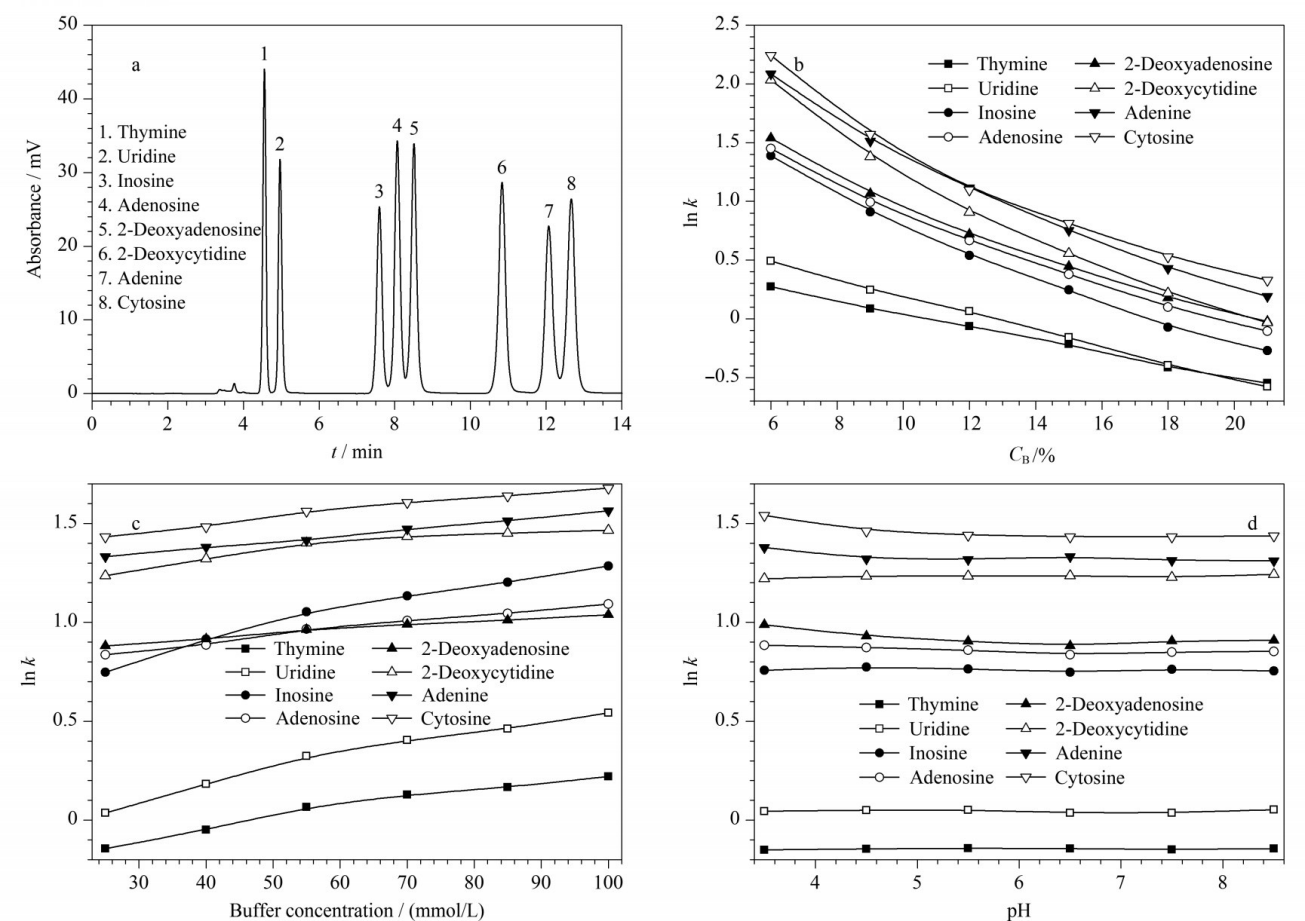
亲水性分析物在亲水模式下的分离性能及保留机制评价

通常在流动相中加入适量的缓冲盐可以有效地改善峰形，提升分离效率。盐浓度对亲水色谱分离性能的影响主要体现在两个方面：一方面，流动相中盐浓度的增加会提升固定相表面“富水层”的厚度，从而提升固定相的亲水分配性能，使分析物在色谱柱上的保留性能增强；另一方面，流动相中盐浓度的增加会对固定相表面的电荷基团形成屏蔽效应，从而削弱固定相与分析物之间的静电引力作用，使分析物在色谱柱上的保留性能减弱。盐浓度对Sil-APA保留性能的影响如图7c所示，Sil-APA对8种碱基/核苷的保留性能随流动相中盐浓度的增加而增加，表明盐浓度的增加提升了Sil-APA固定相的亲水分配性能。

流动相pH值的变化会影响固定相表面电荷的分布以及溶质的质子化程度，从而影响色谱柱的保留性能和分离选择性。Sil-APA柱对亲水化合物的保留性能如图7d所示，在pH为3.5~4.5时，胞嘧啶、腺嘌呤和2-脱氧腺苷的保留性能随pH的增加有较为明显的变化，这是由于随着流动相pH的增加羧基逐渐解离成羧酸根离子，从而削弱Sil-APA固定相与分析物之间的氢键相互作用，导致保留性能有所下降；而羧基的解离又进一步提升了固定相的亲水性。此外，分析物的质子化程度也会随pH的增加而减弱；三者共同影响着Sil-APA固定相对分析物的亲水分配和吸附机制。

#### 2.2.3 离子交换分离模式

Sil-APA固定相表面的羧基除了提供亲水相互作用外，流动相pH值的增加会使其解离成带负电荷的羧酸根，从而在色谱分离过程中为阳离子交换位点通过静电相互作用对阳离子型化合物进行保留^［9］^。以7种有机碱为分析物，分别考察pH和盐浓度对Sil-APA柱保留性能的影响。图8a显示了盐浓度对分析物保留性能的影响。随着盐浓度的升高Sil-APA对7种碱性化合的保留性能逐渐下降，符合离子交换保留特征。相较于普萘洛尔、去甲替林和阿米替林的保留性能随盐浓度出现较大的变化，可可碱、咖啡因、苯胺和对甲基苯胺的变化则较小。这是由于在pH为5.0时，普萘洛尔（p*K*_a_=9.45）、去甲替林（p*K*_a_=9.7）和阿米替林（p*K*_a_=9.3）主要以阳离子化合物的形式存在，其保留以阳离子交换为主，受盐浓度变化的影响更大；可可碱（p*K*_a_=7.89）和咖啡因（p*K*_a_=10.4）虽然以阳离子化合形式存在，但因其极性较大在色谱柱上保留较弱，因此盐浓度的变化对其保留性能的影响并不明显；而苯胺（p*K*_a_=4.63）和对甲基苯胺（p*K*_a_=5.08）则以分子态形态存在，其保留机制以疏水作用为主，几乎不受盐浓度变化的影响。

**图8:**
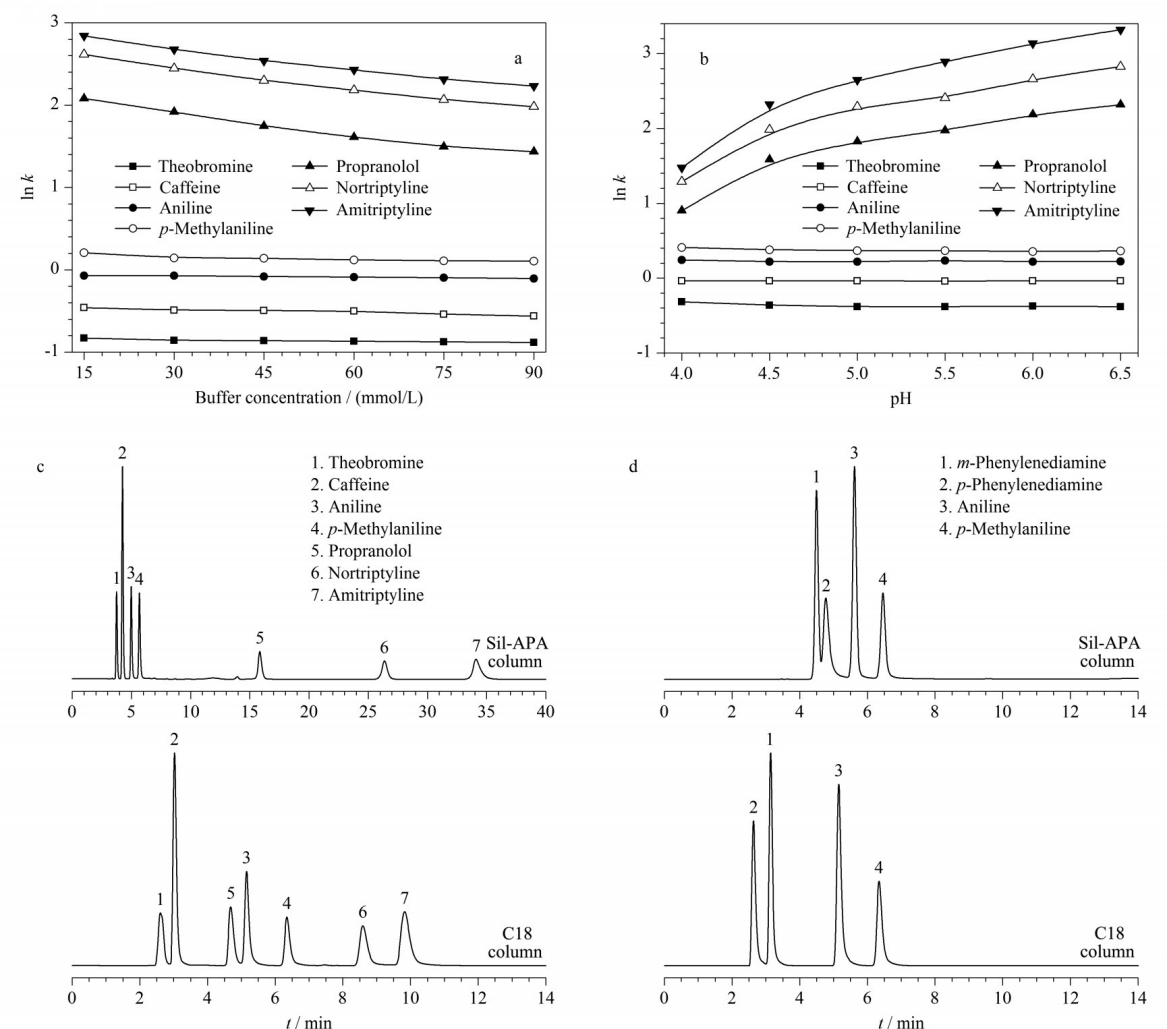
离子交换分离模式下的分离性能及保留机制评价

流动相pH对分析物保留性能的影响如图8b所示，普萘洛尔、去甲替林和阿米替林的保留性能随流动相pH的增加而增加，并且在流动相pH为4.5时出现一个突变点。这是由于松香树脂酸的p*K*_a_约在4.5，此外随着pH的增加，Sil-APA固定相表面羧基的解离程度逐渐增大，固定相与阳离子化合物之间的静电引力作用增强。而在流动相pH为4.5时，可可碱、咖啡因、苯胺和对甲基苯胺的保留性能均有所下降，这由于羧基的解离，削弱了固定相与分析物之间的氢键相互作用。

Sil-APA与C18柱对7种有机碱和4种苯胺类化合物的分离结果如图8c和8d所示。相较于C18柱，除苯胺和对甲基苯胺外，Sil-APA对另外5种有机碱均表现出更强的保留性能和分离性能（见附表5和6）。这是由于在该分离条件下（pH=5.0），Sil-APA固定相表面的羧基已解离成羧酸根，从而增强了固定相的极性和阳离子交换性能，因而对极性化合物（可可碱和咖啡因）和阳离子化合物（普萘洛尔、去甲替林和阿米替林）具有更强的保留性能。由于Sil-APA柱的疏水性弱于C18柱，而苯胺和对甲基苯胺在该色谱条件下（pH=5.0）以分子形态，其保留机制以疏水作用为主，所以Sil-APA柱对苯胺和对甲基苯胺保留性能相对较弱。从图8d可以观察到Sil-APA柱对4种苯胺类化合物不仅具有更强的保留性能，而且其洗脱顺序也与C18柱存在差异。这是由于在该分离条件下（pH=4.5），Sil-APA固定相表面的羧基部分解离，因而固定相能与苯胺类化合物形成静电相互作用，从而增加其保留性能。而Sil-APA柱对对苯二胺（p*K*_a_=4.17）的保留性能强于间苯二胺（p*K*_a_=5.11），则表明氢键相互作用也在苯胺类化合物分离过程中起着重要作用。综上所述，流动相pH的变化会对分析物与固定相之间的氢键和静电相互作用同时造成影响，从而调控Sil-APA色谱柱的保留性能。

### 2.3 分离不同类型混合化合物

Sil-APA固定相表面的氢化菲环和羧基分别作为疏水作用基团和亲水作用基团，在色谱分离过程中能够提供疏水作用、亲水作用和阳离子交换作用，因而在RPLC、HILIC和IEC模式下分别对疏水化合物、亲水化合物及阳离子化合物表现出良好的分离选择性。为考察Sil-APA柱的混合模式分离性能，使用Sil-APA柱对包含中性化合物（甲苯、乙苯、丙苯、芴）、酸性化物（对羟基苯甲酸、对氨基苯甲酸和对甲基苯甲酸）和碱性化合物（对甲基苯胺、对硝基苯胺、普萘洛尔、去甲替林和阿米替林）的混合物进行分离，并与C18柱进行对比，结果如图9所示。相较于C18柱的洗脱顺序按物质的疏水性排列，Sil-APA柱对碱性化合物和芴具有更强的保留性能，这是由于在分离过程中除疏水作用外，Sil-APA柱还可以提供 *π-π* 相互作用和静电相互作用。尽管在该分离条件下 （pH=5.0）羧基解离成羧酸根，Sil-APA固定相与酸性化合物会产生静电斥力，但Sil-APA柱对酸性化合物的保留性能仍强于C18柱，这是由于Sil-APA固定相上存在大量的极性基团，对极性化合物具有更强的保留性能。此外，Sil-APA柱还能在更短的时间内实现12种混合物质的分离。这些结果也表明了Sil-APA固定相对混合分析物具有良好的分离选择性。

**图9:**
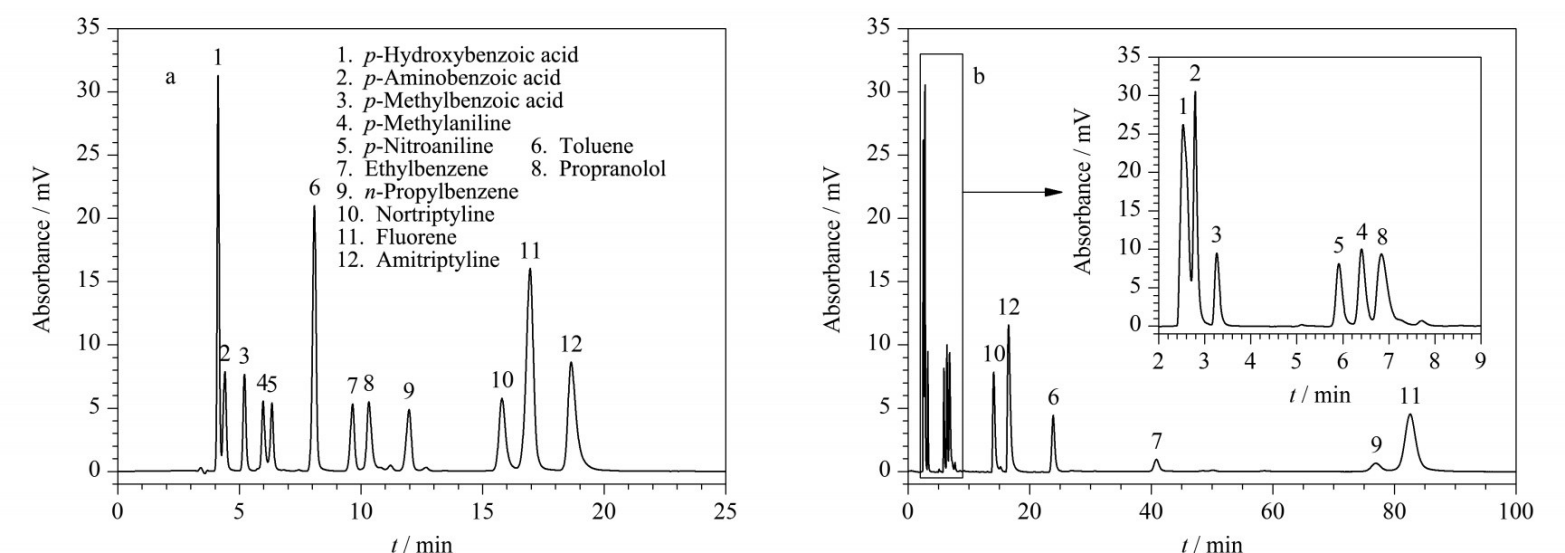
（a） Sil-APA 和 （b） C18柱对混合物的分离色谱图

### 2.4 Sil-APA色谱柱的重复性及重复制备性

重复制备性也是评价Sil-APA色谱柱性能的重要组成部分。分别以12种混合物和8种碱基/核苷为分析物，连续进样10次，通过各物质保留时间的RSD值评价Sil-APA柱的日内稳定性（见附图3a和3b）；以苯和6种PAHs为分析物，通过5天内各物质保留时间的RSD值评价Sil-APA柱的日间稳定性（见附图3c）。12种混合物保留时间的RSD为0.076%~0.356%；8种碱基/核苷保留时间的RSD为0.117%~0.243%；苯和6种PAHs保留时间的RSD为 0.05%~0.193%，表明该色谱柱在不同的分离模式下均具有良好的重复性和稳定性。为了评价 Sil-APA色谱柱的重复制备性，重新制备了3批色谱填料并将其装填成柱，考察了12种混合物在不同批次Sil-APA色谱柱上的分离情况。从附图3d可以观察到12种化合物在4根不同批次的色谱柱上均实现基线分离，且洗脱顺序一致；12种化合物在4根不同批次的色谱柱上保留时间的RSD为0.498%~2.806%。该结果不仅表明Sil-APA色谱柱具有良好的稳定性和可重复制备性，也表明利用丙烯海松酸制备混合模式色谱固定相的设计策略是可行的。

## 3 结论

本文以丙烯海松酸为功能单体，通过开环酯化法将其键合到硅胶表面，制备得到一种新型的松香基混合模式色谱固定相Sil-APA。分别采用疏水和亲水化合物对Sil-APA的分离性能和保留机制进行考察，结果表明所制备的固定相具有良好的反相和亲水保留性能；Sil-APA对碱性化合物的良好分离性能表明该固定相还具有弱阳离子交换性能。Sil-APA固定相表面的羧基，在提高其亲水性使其能在更短时间内实现疏水性化合物分离的同时；还能提供氢键和 *π-π* 相互作用，从而对黄酮和多环芳烃类化合物具有更好的分离选择性。此外，Sil-APA柱在不同分离模式下均表现出良好的重复性和稳定性；并且不同批次的Sil-APA柱对混合样品均具有良好的分离性能，这不仅表明Sil-APA色谱柱有望适用于复杂样品的分析，还表明Sil-APA固定相的设计和制备策略是可行的。
